# Projecting the future of dengue under climate change scenarios: Progress, uncertainties and research needs

**DOI:** 10.1371/journal.pntd.0008118

**Published:** 2020-03-02

**Authors:** Zhiwei Xu, Hilary Bambrick, Francesca D. Frentiu, Gregor Devine, Laith Yakob, Gail Williams, Wenbiao Hu

**Affiliations:** 1 School of Public Health and Social Work, Queensland University of Technology, Brisbane, Australia; 2 Institute of Health and Biomedical Innovation, Queensland University of Technology, Brisbane, Australia; 3 School of Biomedical Sciences, Queensland University of Technology, Brisbane, Australia; 4 Mosquito Control Laboratory, QIMR Berghofer Medical Research Institute, Brisbane, Australia; 5 Department of Disease Control, London School of Hygiene and Tropical Medicine, London, United Kingdom; 6 School of Public Health, University of Queensland, Brisbane, Australia; Mahidol University, THAILAND

## Abstract

**Background:**

Dengue is a mosquito-borne viral disease and its transmission is closely linked to climate. We aimed to review available information on the projection of dengue in the future under climate change scenarios.

**Methods:**

Using five databases (PubMed, ProQuest, ScienceDirect, Scopus and Web of Science), a systematic review was conducted to retrieve all articles from database inception to 30^th^ June 2019 which projected the future of dengue under climate change scenarios. In this review, “the future of dengue” refers to disease burden of dengue, epidemic potential of dengue cases, geographical distribution of dengue cases, and population exposed to climatically suitable areas of dengue.

**Results:**

Sixteen studies fulfilled the inclusion criteria, and five of them projected a global dengue future. Most studies reported an increase in disease burden, a wider spatial distribution of dengue cases or more people exposed to climatically suitable areas of dengue as climate change proceeds. The years 1961–1990 and 2050 were the most commonly used baseline and projection periods, respectively. Multiple climate change scenarios introduced by the Intergovernmental Panel on Climate Change (IPCC), including B1, A1B, and A2, as well as Representative Concentration Pathway 2.6 (RCP2.6), RCP4.5, RCP6.0 and RCP8.5, were most widely employed. Instead of projecting the future number of dengue cases, there is a growing consensus on using “population exposed to climatically suitable areas for dengue” or “epidemic potential of dengue cases” as the outcome variable. Future studies exploring non-climatic drivers which determine the presence/absence of dengue vectors, and identifying the pivotal factors triggering the transmission of dengue in those climatically suitable areas would help yield a more accurate projection for dengue in the future.

**Conclusions:**

Projecting the future of dengue requires a systematic consideration of assumptions and uncertainties, which will facilitate the development of tailored climate change adaptation strategies to manage dengue.

## Author summary

Dengue is the most important arboviral disease globally, and the transmission of dengue is closely linked to climate. This review assembled all existing studies which have quantified the impact of climate change on dengue under climate change scenarios. We observed that most studies reported an increase in disease burden, a wider spatial distribution of dengue cases or more people exposed to climatically suitable areas of dengue as climate change proceeds. The years 1961–1990 and 2050 were the most commonly used baseline and projection periods, respectively. Multiple climate change scenarios introduced by the Intergovernmental Panel on Climate Change (IPCC), including B1, A1B, and A2, as well as Representative Concentration Pathway 2.6 (RCP2.6), RCP4.5, RCP6.0 and RCP8.5, were most widely employed. Instead of projecting the future number of dengue cases, there is a growing consensus on using “population exposed to climatically suitable areas for dengue” or “epidemic potential of dengue cases” as the outcome variable. Future studies exploring non-climatic drivers which determine the presence/absence of dengue vectors, and identifying the pivotal factors triggering the transmission of dengue in those climatically suitable areas would help yield a more accurate projection for dengue in the future.

## Introduction

Dengue is the most important arboviral disease globally, with an estimated 390 million dengue infections per year [1] and causes an enormous economic burden to governments and households [2]. The number of deaths due to dengue is increasing in recent years [3]. It has been reported that over 3.9 billion people in 128 countries are at risk of dengue infection [4]. Climatic factors affect the occurrence of dengue by impacting on the life cycle and transmission of dengue viruses, as well as the growth and survival of dengue vectors (i.e., *Aedes aegypti* and *Aedes albopictus*) [5]. Hence, the association between climatic factors and dengue has been widely researched [5]. For example, Li et al. have observed that climate-driven variation in mosquito density could predict the spatiotemporal dynamics of dengue in China [6].

Climate change is occurring and affecting human health and wellbeing [7]. As climate change continues, the global surface temperature will increase and the pattern of rainfall will change [8], which will affect the environmental suitability for the growth and survival of dengue viruses and mosquitoes, and may subsequently change the burdens of dengue globally, nationally, and locally. There has been an increasing number of studies projecting the future disease burden of dengue, epidemic potential of dengue cases, geographical distribution of dengue cases, or population exposed to climatically suitable areas of dengue under climate change scenarios [9–20]. Nevertheless, appreciable heterogeneity exists in these projections in terms of modelling approaches used and future scenarios adopted. Messina et al. have assembled the existing studies projecting the global future of dengue under climate change scenarios and have discussed the popular methods used in these studies [21]. However, regional or local studies were not included in the review of Messina et al.

In the present study, we attempted to review all available studies which projected the future disease burden of dengue, epidemic potential of dengue cases, geographical distribution of dengue cases, or population exposed to climatically suitable areas of dengue (hereinafter called “the future of dengue”) under climate change scenarios, identify the uncertainties in this field and propose the future research needs.

## Methods

### Data sources

Empirical studies projecting the future of dengue under climate change scenarios published up to 30^th^ June 2019 were retrieved using PubMed, ProQuest, ScienceDirect, Scopus and Web of Science. The references of the identified papers were examined visually to make sure that all eligible papers were included in the final review.

### Inclusion criteria

We restricted the search to peer-reviewed papers written in English. Our primary search used the following U.S. National Library of Medicine's Medical Subject Headings (MeSH terms) and keywords: “dengue”, “climate”, “prediction”, “projection”, “forecast”, and “predicting”. Eligibility included those papers which projected the future disease burden of dengue, epidemic potential of dengue cases, geographical distribution of dengue cases, or population exposed to climatically suitable areas of dengue under climate change scenarios around the globe or in one country/city using at least one climate change scenario. Climate change scenario is defined as a description of the future change in climate under concrete assumptions on the future growth of greenhouse gas (GHG) and on other factors which may impact future climate. The most widely used climate change scenarios are those developed by the Intergovernmental Panel on Climate Change (IPCC). In the IPCC’s Fourth Assessment Report, three climate change scenarios detailed in the Special Report on Emissions Scenarios (SRES) were B1, A1B, and A2 [22]. In the IPCC’s Fifth Assessment Report, the emissions scenarios were called Representative Concentration Pathways (RCPs), and the four RCPs were RCP2.6 (low emission scenario), RCP4.5 and 6.0 (intermediate emission scenarios), and RCP8.5 (high emission scenario) [8]. Although the presence of vectors is essential for the occurrence of dengue cases, published papers solely projecting the future distribution of dengue mosquitoes were not included in this review because the main outcome-of-interest of this review is human health.

## Results

**Fig 1:**
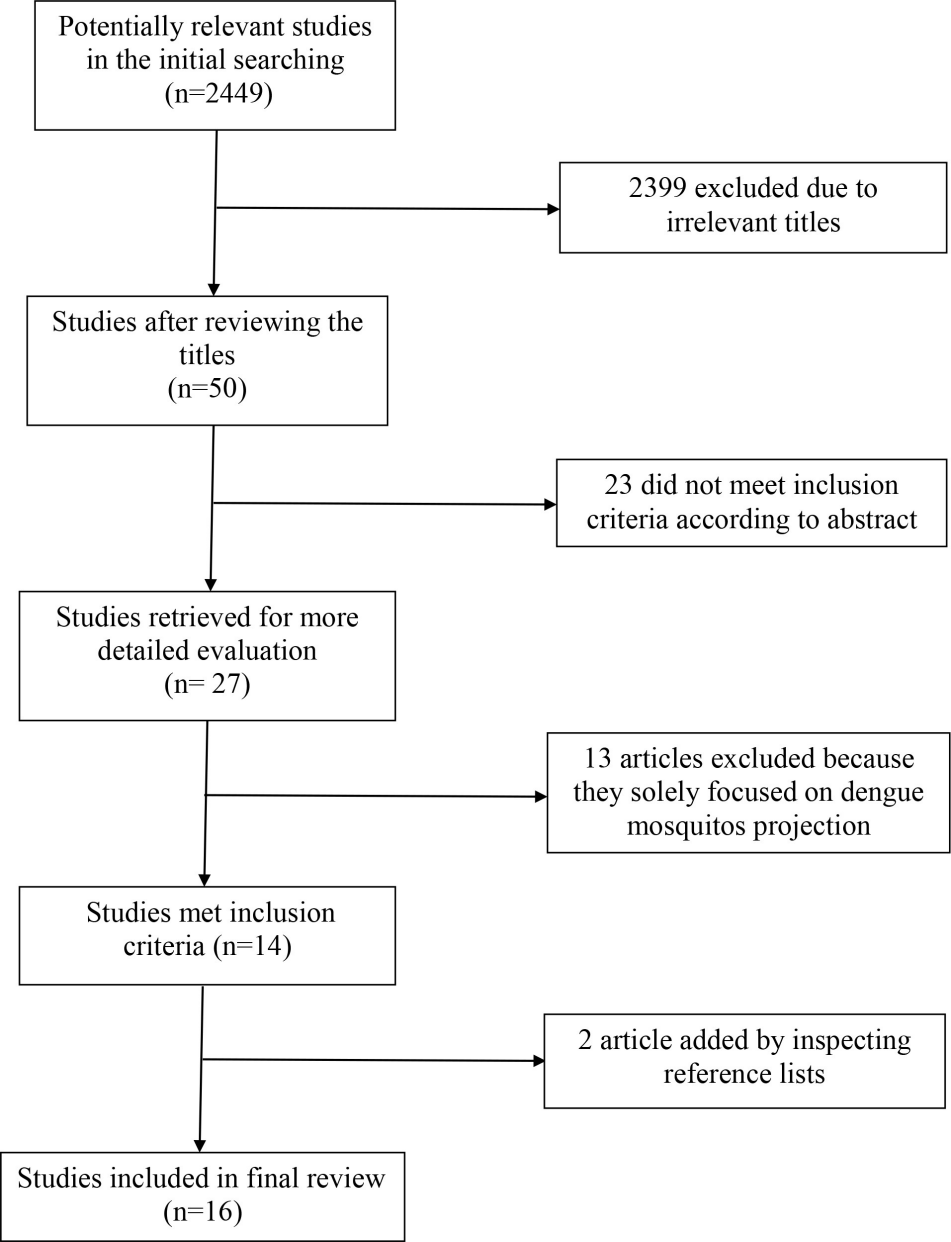
The flow chart of literature selection process.

We identified 2,449 articles in the initial search, and 16 of them entered the final review according to the inclusion criteria (Fig 1). The specific characteristics of these 16 articles are presented in Table 1.

**Table 1:** Characteristics of the studies projecting the burden or geographical distribution of dengue under climate change scenarios.

Study	Setting	Baseline period	Projection period	Climate change scenarios	Spatial resolution	Modelling approach	Outcomes
Acharya et al. 2018	Nepal	1950–2000	2050 and 2070	RCP2.6, RCP6.0 and RCP8.5	30 arc second	Mechanistic model	Population exposed to climatically suitable areas of dengue
Bambrick et al. 2009	Australia	1961–1990	2100	Four climate change scenarios produced by CSIRO	Not given	Correlative model	Distribution of dengue cases and people living in regions of high risk of dengue transmission
Banu et al. 2014	Dhaka, Bangladesh	2000–2010	2100	Monthly temperature increases by 1, 2 and 3.3°C in 2100 relative to 2010	Not given	Correlative model	Annual number of dengue cases
Bouzid et al. 2014	Europe	1961–1990	2011–2040, 2041–2070, 2071–2100	A1B	10 km * 10 km	Correlative model	Number and geographical distribution of dengue cases
Butterworth et al. 2017	23 locations of the US	1961–1990	2045–2065	A1B	1.3^o^ – 3.9^o^	Mechanistic model	Number of dengue cases
Study	Setting	Baseline period	Projection period	Climate change scenarios	Spatial resolution	Modelling approach	Outcomes
Colon-Gonzalez et al. 2013	Mexico	1970–1999	2030, 2050, and 2080	A1B, A2 and B1	Not given	Correlative model	The average value and distribution of annual dengue incidence
Fan et al. 2019	China	1981–2016	2020, 2030, 2050 and 2100	RCP2.6, RCP4.5, RCP6.0 and RCP8.5	0.5^o^ * 0.5^o^	Mechanistic model	Distribution of dengue cases
Lee et al. 2018	Korea	2012–2016	2020–2099	RCP2.6, RCP4.5, RCP6.0 and RCP8.5	Not given	Mechanistic model	Potential risk of dengue outbreaks
Li et al. 2017	Guangzhou, China	1998–2014	2020–2070	RCP2.6 and RCP8.5	Not given	Correlative model	Number of dengue cases
Liu-Helmersson et al. 2016	10 European cities	1901–1930, 1984–2013	2070–2099	RCP2.6, RCP4.5, RCP6.0 and RCP8.5	0.25^o^ * 0.25^o^	Mechanistic model	The seasonal peak and time window for dengue epidemic potential
Williams et al. 2016	Four cities (Brisbane, Cairns, Rockhampton, and Townsville) in Queensland, Australia.	1990–2011	2046–2064	A2 and B1	Not given	Mechanistic model	Probability of dengue outbreaks and epidemic potential
Study	Setting	Baseline period	Projection period	Climate change scenarios	Spatial resolution	Modelling approach	Outcomes
Astrom et al. 2012	Globe	1961–1990	2050	A1B	0.5^o^ * 0.5^o^	Correlative model	Population at risk of dengue and its distribution
Hales et al. 2002	Globe	1961–1990	2050 and 2080	IS92a and IS92f	0.5^o^ * 0.5^o^	Correlative model	Population at risk of dengue
Martens et al. 1997	Globe	1931–1980	2050	GFDL89, UKTR, and ECHAM1-A	Not given	Mechanistic model	Epidemic potential of dengue cases
Messina et al. 2019	Globe	1960–2015	2020, 2050 and 2080	RCP4.5, RCP6.0 and RCP8.5	5 km * 5 km	Mechanistic model	Environmental suitability for dengue virus and population at risk of dengue
Patz et al. 1998	Globe	1931–1980	2050	Three GCMs	250 km horizontally and 1 km vertically	Mechanistic model	Dengue average annual epidemic potential

### Local, national or regional studies

Eleven of the 16 studies included in the final review projected the future of dengue at the local, national, or regional level (Table 1). Specifically, the research settings of these studies were Australia [11, 20], Bangladesh [12], China [15, 17], Europe [23, 24], Korea [25], Mexico [14], Nepal [9], and the US [13]. These studies were largely heterogeneous in five key aspects. First, the baseline period used varied: three studies used 1961–1990 as the baseline period [11, 13, 23], but the baseline periods used in the other eight studies varied. The inconsistency in the baseline period employed in different studies renders it difficult to directly compare the projection results across these studies. Second, the projection period also varied among studies: five studies used one year (e.g., 2100) or a couple of different years (e.g., 2050 and 2070) as the projection period [9, 11, 12, 14, 15], and the other six studies used a consecutive period of time (e.g., 2070–2090) as the projection period [13, 17, 20, 23–25]. The formation of a wide consensus on the use of projection periods (e.g. short-term (2030), middle-term (2050) and long-term (2100)) would facilitate the comparison of future study results. Third, the climate change scenarios used varied: four studies conducted in Australia [20], Europe [23], Mexico [14], and the US [13] used A1B, A2 and/or B1 as the climate change scenarios, and five studies conducted in China [15, 17], Europe [24], Korea [25] and Nepal [9] used RCPs to project the future of dengue. The study by Bambrick et al. used the climate change scenarios produced by CSIRO (the Commonwealth Scientific and Industrial Research Organisation of Australia) [11] and the study of Banu et al. used the climate change scenarios assuming that the monthly temperature in 2100 will increase by 1, 2 or 3.3°C relative to 2010 [12]. Fourth, the modelling approach used: there are generally two types of models used in projecting the future of dengue, i.e., mechanistic model and correlative model [21]. The strengths and limitations of these two modelling approaches can be found in the previous review papers [21, 26]. In the 11 studies which projected the future of dengue locally, nationally, or regionally, six used mechanistic modelling approach [9, 13, 15, 20, 24, 25], and the other five used correlative modelling approach [11, 12, 14, 17, 23]. Last, the outcome variable also differed: five studies projected the future number of dengue cases [12–14, 17, 23], four studies projected the future spatial distribution of dengue cases/incidence [11, 14, 15, 23], two studies projected the future population exposed to climatically suitable areas of dengue or future population living in regions of high risk of dengue transmission [9, 11], and three studies projected the future dengue epidemic potential [20, 24, 25].

### Global studies

At 30^th^ June 2019, there were five studies which projected the future of dengue at the global scale (Table 1) [10, 16, 18, 19, 27]. Interestingly, the period 1961–1990 was also used as the baseline period in two of these five studies [10, 16], and 1931–1980 was used as the baseline period in another two studies [18, 19]. Regarding the projection period, all of the five studies used 2050 or a couple of years including 2050 and 2080 as the projection period to project the future of dengue globally. The climate change scenarios employed in these global studies varied from one to another, and, as some studies were conducted before SRES or RCPs were introduced, they used some older climate change scenarios (e.g., GFDL89 [18]). In terms of the outcome variables used, three of these studies projected the future global population at risk of dengue and its spatial distribution [10, 16, 27], two projected the spatial pattern of dengue epidemic potential globally [18, 19], and one projected the spatial pattern of environmental suitability for dengue virus globally [27].

## Discussion

### Progress

As the transmission of dengue involves dengue viruses, vectors, and susceptible people, to understand the precise relationship between climate and dengue transmission is not a trivial task [6, 28]. Further, projecting the future of dengue under climate change scenarios requires not just a good understanding of the association between climate and dengue but also comprehensive knowledge on future changes in climate and other factors (e.g. demographic change). Nevertheless, much progress has been made in this field. First, there is a growing consensus on using “population exposed to climatically suitable areas of dengue” or “epidemic potential of dengue cases” as the outcome variable in the projection [9, 24, 27], instead of projecting the absolute number of future dengue cases. Second, with the advent of the multiple climate change scenarios introduced by IPCC covering the “best case scenario” and the possible “worst case scenario” [8, 22], the selection of climate change scenarios has become more consistent across different studies. Third, the presence of dengue vectors is pivotal for the transmission of dengue, but projecting the distribution of dengue vectors is challenging partially due to the unavailability of rich data on the present distribution of dengue vectors. Nevertheless, there have been a few attempts which incorporated findings on the current and future distributions of dengue vectors into the projection of dengue future [29–31]. Kraemer et al. have investigated the past and projected future spread of *A*. *aegypti* and *A*. *albopictus* globally [30], and based on this work, Messina et al. have presented the current and future global population at risk of dengue [27].

### Uncertainties

Despite the progresses made in the projection of dengue future, many uncertainties remain to be resolved. First, sociodemographic factors play an appreciable role in the transmission of dengue, and incorporating sociodemographic factors in the projection of dengue future remains a challenge. A salient example is the relationship between travel and the transmission of dengue [32–34]. In 2016, there were more than 1.2 billion international tourists and this number is still growing [35], raising concerns about the appreciable role that travel (particularly international travel [36]) may play in the future transmission of dengue. Second, increasing temperature has been widely used as the indicator of climate change in the prior studies projecting the future of dengue, with rainfall and humidity being under-researched. Hales et al. reported that vapour pressure, an index which incorporates temperature and humidity, is the climate indicator which predicts the presence of dengue most accurately [16]. However, the associations of different climatic factors with the transmission of dengue are complex and sometimes behave in a non-linear manner [5, 37]. Third, the crucial drivers behind the presence or absence of dengue vectors include, but are not limited to, climate or vector-control programs [38], and other fundamental drivers remain to be unveiled. Fourth, why dengue transmission occurs in some regions with ideal environment and vectors, but not in other similar regions, remains mysterious.

### Future research needs

Accurately projecting the future of dengue under the context of climate change would help governments and public health officials take timely and pre-emptive actions to protect the public from dengue in the future. There are several knowledge gaps that need to be filled in this field. First, incorporating the most important sociodemographic factors (e.g., travel and demographic change) into the projections would yield a more accurate estimate of dengue future [25]. Second, in some regions, temperature might not be the most significant climatic factor associated with the transmission of dengue [39, 40]. Identifying the locally important climatic factor and conducting precise projection at the local level is warranted. Third, it is of great significance to explore the non-climatic drivers behind the presence of *A*. *aegypti and A*. *albopictus*, and also to identify the crucial factors triggering the transmission of dengue in those climatically suitable regions. Fourth, some dengue control strategies may be effective in curbing its spread in some areas [41]. As more evidence of their effectiveness accumulates (e.g., *Wolbachia* [42, 43]), such strategies need to be taken into account in dengue projections as some high risk regions for transmission may become low risk due to vector control capacity [44]. Fifth, routine communication between the research community and policy makers on the local key drivers of dengue transmission is still deficient, calling for concerted efforts to be made in the future.

### Limitations of this review

Several limitations of this review should be acknowledged. First, the different outcomes used in the existing studies projecting the future of dengue under climate change scenarios restricted us to quantitatively pool the findings. Second, understanding the future distribution of dengue vectors is an essential step in adequately understanding the future of dengue, but those studies solely projecting the future distribution of dengue vectors under climate change scenarios were not included in this review due to the focus of this review being on human health. Third, specific methodological issues in projecting the future of dengue (e.g., proper control of confounders) worth exploring but were not comprehensively elucidated in this review because some published review papers have discussed these issues to some extent.

### Conclusion

As climate change proceeds, population exposed to areas with suitable environment for the transmission of dengue may change. There is an increasing number of studies which projected the future of dengue under climate change scenarios. Identifying the non-climatic drivers behind the presence/absence of dengue vectors and the pivotal factors triggering the transmission of dengue in those climatically suitable areas is an important next step. In addition to future projections accounting for alternative climate change scenarios, benefit would come from considering different control scenarios (e.g., programs incorporating Wolbachia). This would not only improve projection realism but would also act as an impetus for establishing researchers and policy makers’ consensus on provisions to mitigate future dengue.

## Supporting information

S1 Checklist PRISMA checklist. (DOC)

S1 Flowchart PRISMA flowchart. (DOC)

## References

[1] Bhatt S, Gething PW, Brady OJ, Messina JP, Farlow AW, Moyes CL, et al The global distribution and burden of dengue. Nature. 2013;496:504–7. 10.1038/nature12060 23563266 PMC3651993

[2] Shepard DS, Undurraga EA, Halasa YA, Stanaway JD. The global economic burden of dengue: a systematic analysis. The Lancet Infectious Diseases. 2016;16(8):935–41. 10.1016/S1473-3099(16)00146-8 27091092

[3] Ferguson NM. Challenges and opportunities in controlling mosquito-borne infections. Nature. 2018;559(7715):490–7. 10.1038/s41586-018-0318-5 30046071

[4] Brady OJ, Gething PW, Bhatt S, Messina JP, Brownstein JS, Hoen AG, et al Refining the global spatial limits of dengue virus transmission by evidence-based consensus. PLoS neglected tropical diseases. 2012;6(8):e1760–e. 10.1371/journal.pntd.0001760 . 22880140 PMC3413714

[5] Morin C, Comrie A, Ernst K. Climate and dengue transmission: evidence and implications. Environmental Health Perspectives 2013;121(11–12):1264–72. 10.1289/ehp.1306556 24058050 PMC3855512

[6] Li R, Xu L, Bjørnstad ON, Liu K, Song T, Chen A, et al Climate-driven variation in mosquito density predicts the spatiotemporal dynamics of dengue. Proceedings of the National Academy of Sciences of the United States of America. 2019;116(9):3624–9. Epub 02/11. 10.1073/pnas.1806094116 . 30808752 PMC6397594

[7] Haines A, Ebi K. The Imperative for Climate Action to Protect Health. New England Journal of Medicine. 2019;380(3):263–73. 10.1056/NEJMra1807873 . 30650330

[8] IPCC. Climate Change 2014: Synthesis Report. Contribution of Working Groups I, II and III to the Fifth Assessment Report of the Intergovenmental Panel on Climate Change. Geneva, Switzerland 2014.

[9] Acharya BK, Cao C, Xu M, Khanal L, Naeem S, Pandit S. Present and Future of Dengue Fever in Nepal: Mapping Climatic Suitability by Ecological Niche Model. International journal of environmental research and public health. 2018;15(2):187 10.3390/ijerph15020187 . 29360797 PMC5857046

[10] Åström C, Rocklöv J, Hales S, Béguin A, Louis V, Sauerborn R. Potential Distribution of Dengue Fever Under Scenarios of Climate Change and Economic Development. EcoHealth. 2012;9(4):448–54. 10.1007/s10393-012-0808-0 23408100

[11] Bambrick HJ, Woodruff RE, Hanigan IC. Climate change could threaten blood supply by altering the distribution of vector-borne disease: an Australian case-study. Global health action. 2009;2:10.3402/gha.v2i0.2059. 10.3402/gha.v2i0.2059 . 20052315 PMC2802100

[12] Banu S, Hu W, Guo Y, Hurst C, Tong S. Projecting the impact of climate change on dengue transmission in Dhaka, Bangladesh. Environment International. 2014;63:137–42. 10.1016/j.envint.2013.11.002 24291765

[13] Butterworth MK, Morin CW, Comrie AC. An Analysis of the Potential Impact of Climate Change on Dengue Transmission in the Southeastern United States. Environmental health perspectives. 2017;125(4):579–85. Epub 10/07. 10.1289/EHP218 . 27713106 PMC5381975

[14] Colón-González FJ, Fezzi C, Lake IR, Hunter PR. The Effects of Weather and Climate Change on Dengue. PLOS Neglected Tropical Diseases. 2013;7(11):e2503 10.1371/journal.pntd.0002503 24244765 PMC3828158

[15] Fan J-C, Liu Q-Y. Potential impacts of climate change on dengue fever distribution using RCP scenarios in China. Advances in Climate Change Research. 2019;10(1):1–8. 10.1016/j.accre.2019.03.006.

[16] Hales S, de Wet N, Maindonald J, Woodward A. Potential effect of population and climate changes on global distribution of dengue fever: an empirical model. The Lancet. 2002;360(9336):830–4. 10.1016/S0140-6736(02)09964-6. 12243917

[17] Li C, Wang X, Wu X, Liu J, Ji D, Du J. Modeling and projection of dengue fever cases in Guangzhou based on variation of weather factors. Science of The Total Environment. 2017;605–606:867–73. 10.1016/j.scitotenv.2017.06.181. 28683431

[18] Martens WJM, Jetten TH, Focks DA. SENSITIVITY OF MALARIA, SCHISTOSOMIASIS AND DENGUE TO GLOBAL WARMING. Climatic Change. 1997;35(2):145–56. 10.1023/A:1005365413932

[19] Patz JA, Martens WJ, Focks DA, Jetten TH. Dengue fever epidemic potential as projected by general circulation models of global climate change. Environmental health perspectives. 1998;106(3):147–53. 10.1289/ehp.98106147 . 9452414 PMC1533051

[20] Williams CR, Mincham G, Faddy H, Viennet E, Ritchie SA, Harley D. Projections of increased and decreased dengue incidence under climate change. Epidemiology and Infection. 2016;144(14):3091–100. Epub 07/26. 10.1017/S095026881600162X 27457660 PMC9150423

[21] Messina JP, Brady OJ, Pigott DM, Golding N, Kraemer MUG, Scott TW, et al The many projected futures of dengue. Nature Reviews Microbiology. 2015;13:230 10.1038/nrmicro3430 25730702

[22] IPCC. Climate Change 2007: Synthesis Report. Contribution of Working Groups I, II and III to the Fourth Assessment Report of the Intergovernmental Panel on Climate Change. Geneva, Switzerland 2007.

[23] Bouzid M, Colón-González FJ, Lung T, Lake IR, Hunter PR. Climate change and the emergence of vector-borne diseases in Europe: case study of dengue fever. BMC Public Health. 2014;14(1):781 10.1186/1471-2458-14-781 25149418 PMC4143568

[24] Liu-Helmersson J, Quam M, Wilder-Smith A, Stenlund H, Ebi K, Massad E, et al Climate Change and Aedes Vectors: 21st Century Projections for Dengue Transmission in Europe. EBioMedicine. 2016;7:267–77. 10.1016/j.ebiom.2016.03.046 . 27322480 PMC4909611

[25] Lee H, Kim JE, Lee S, Lee CH. Potential effects of climate change on dengue transmission dynamics in Korea. PLOS ONE. 2018;13(6):e0199205 10.1371/journal.pone.0199205 29953493 PMC6023222

[26] Tjaden NB, Caminade C, Beierkuhnlein C, Thomas SM. Mosquito-Borne Diseases: Advances in Modelling Climate-Change Impacts. Trends in Parasitology. 2018;34(3):227–45. 10.1016/j.pt.2017.11.006 29229233

[27] Messina JP, Brady OJ, Golding N, Kraemer MUG, Wint GRW, Ray SE, et al The current and future global distribution and population at risk of dengue. Nature Microbiology. 2019 10.1038/s41564-019-0476-8 31182801 PMC6784886

[28] Xu L, Stige LC, Chan K-S, Zhou J, Yang J, Sang S, et al Climate variation drives dengue dynamics. Proceedings of the National Academy of Sciences. 2017;114(1):113–8. 10.1073/pnas.1618558114 27940911 PMC5224358

[29] Ducheyne E, Tran Minh NN, Haddad N, Bryssinckx W, Buliva E, Simard F, et al Current and future distribution of Aedes aegypti and Aedes albopictus (Diptera: Culicidae) in WHO Eastern Mediterranean Region. Int J Health Geogr. 2018;17(1):4–. 10.1186/s12942-018-0125-0 . 29444675 PMC5813415

[30] Kraemer MUG, Reiner RC, Brady OJ, Messina JP, Gilbert M, Pigott DM, et al Past and future spread of the arbovirus vectors Aedes aegypti and Aedes albopictus. Nature Microbiology. 2019;4(5):854–63. 10.1038/s41564-019-0376-y 30833735 PMC6522366

[31] Ryan SJ, Carlson CJ, Mordecai EA, Johnson LR. Global expansion and redistribution of Aedes-borne virus transmission risk with climate change. PLOS Neglected Tropical Diseases. 2019;13(3):e0007213 10.1371/journal.pntd.0007213 30921321 PMC6438455

[32] Choe Y-J, Choe S-A, Cho S-I. Importation of travel-related infectious diseases is increasing in South Korea: An analysis of salmonellosis, shigellosis, malaria, and dengue surveillance data. Travel Medicine and Infectious Disease. 2017;19:22–7. 10.1016/j.tmaid.2017.09.003 28919170 PMC7110683

[33] Findlater A, Moineddin R, Kain D, Yang J, Wang X, Lai S, et al The use of air travel data for predicting dengue importation to China: A modelling study. Travel Medicine and Infectious Disease. 2019 10.1016/j.tmaid.2019.07.002. 31284067

[34] Tian H, Sun Z, Faria NR, Yang J, Cazelles B, Huang S, et al Increasing airline travel may facilitate co-circulation of multiple dengue virus serotypes in Asia. PLoS neglected tropical diseases. 2017;11(8):e0005694–e. 10.1371/journal.pntd.0005694 . 28771468 PMC5542384

[35] UNTWO. Compendium of Tourism Statistics, Data 2011–2015 2017.

[36] Xu Z, Bambrick H, Yakob L, Devine G, Frentiu FD, Marina R, et al Using dengue epidemics and local weather in Bali, Indonesia to predict imported dengue in Australia. Environmental Research. 2019;175:213–20. 10.1016/j.envres.2019.05.021 31136953

[37] Xu Z, Bambrick H, Yakob L, Devine G, Lu J, Frentiu FD, et al Spatiotemporal patterns and climatic drivers of severe dengue in Thailand. Science of The Total Environment. 2019;656:889–901. 10.1016/j.scitotenv.2018.11.395 30625675

[38] Russell RC, Currie BJ, Lindsay MD, Mackenzie JS, Ritchie SA, Whelan PI. Dengue and climate change in Australia: predictions for the future should incorporate knowledge from the past. Medical Journal of Australia. 2009;190(5):265–8. 10.5694/j.1326-5377.2009.tb02393.x 19296793

[39] Wang X, Tang S, Wu J, Xiao Y, Cheke RA. A combination of climatic conditions determines major within-season dengue outbreaks in Guangdong Province, China. Parasites & vectors. 2019;12(1):45–. 10.1186/s13071-019-3295-0 . 30665469 PMC6341621

[40] Benedum CM, Seidahmed OME, Eltahir EAB, Markuzon N. Statistical modeling of the effect of rainfall flushing on dengue transmission in Singapore. PLoS neglected tropical diseases. 2018;12(12):e0006935–e. 10.1371/journal.pntd.0006935 . 30521523 PMC6283346

[41] O'Neill SL, Ryan PA, Turley AP, Wilson G, Retzki K, Iturbe-Ormaetxe I, et al Scaled deployment of Wolbachia to protect the community from dengue and other Aedes transmitted arboviruses. Gates open research. 2018;2:36–. 10.12688/gatesopenres.12844.3 . 30596205 PMC6305154

[42] Dorigatti I, McCormack C, Nedjati-Gilani G, Ferguson NM. Using Wolbachia for dengue control: Insights from modelling. Trends in parasitology. 2018;34(2):102–13. Epub 11/25. 10.1016/j.pt.2017.11.002 . 29183717 PMC5807169

[43] Walker T, Johnson PH, Moreira LA, Iturbe-Ormaetxe I, Frentiu FD, McMeniman CJ, et al The wMel Wolbachia strain blocks dengue and invades caged Aedes aegypti populations. Nature. 2011;476(7361):450–3. 10.1038/nature10355 21866159

[44] Zheng X, Zhang D, Li Y, Yang C, Wu Y, Liang X, et al Incompatible and sterile insect techniques combined eliminate mosquitoes. Nature. 2019;572(7767):56–61. 10.1038/s41586-019-1407-9 31316207

