# Supplementary material for: Projecting the future of dengue under climate change scenarios: Progress, uncertainties and research needs
Source: PLoS Negl Trop Dis. 2020 Mar 2;14(3):e0008118. doi: 10.1371/journal.pntd.0008118 (PMC7067491; doi:10.1371/journal.pntd.0008118)
Supplement: S1 Flowchart — (DOC) [file pntd.0008118.s002.doc]

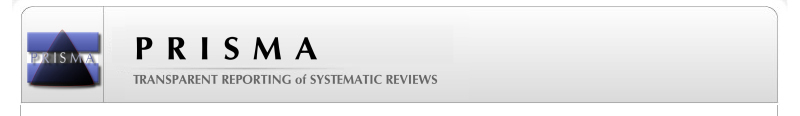
**PRISMA 2009 Flow Diagram**

**Screening**

**Included**

**Eligibility**

**Identification**

Records identified through database searching
(n =3258)

Records after duplicates removed
(n =2449)

Records screened
(n =2449)

Records excluded due to irrelevant titles
(n =2399)

Full-text articles assessed for eligibility
(n =50)

Full-text articles excluded because they did not meet the inclusion criteria or because they solely focused on dengue mosquito projection
(n =34)

Studies included in qualitative synthesis
(n =16)
