# Supplementary material for: Projecting the future of dengue under climate change scenarios: Progress, uncertainties and research needs
Source: PLoS Negl Trop Dis. 2020 Mar 2;14(3):e0008118. doi: 10.1371/journal.pntd.0008118 (PMC7067491; doi:10.1371/journal.pntd.0008118)
Supplement: Attachment — Submitted filename: Response to reviewers.docx [file pntd.0008118.s003.docx]

Dear Reviewers,

Thanks very much for the valuable comments on our manuscript. We have revised the manuscript accordingly and all revisions have been marked red.

**Methods**
**Reviewer #1: The objectives were clearly stated. The study methods were described clearly. Authors had performed a comprehensive search.**

Response: Thanks for the comment.
**Reviewer #2: Authors should explain why papers solely projecting the future distribution of dengue mosquitoes were not included in this review.**

Response: Thanks for the comment. We have clarified this in the revision (lines 134-136).
--------------------

**Results
Reviewer #1: The analysis was organised neatly and presented clearly.**
Response: Thanks.
**Reviewer #2: (No Response)**
--------------------

**Conclusions**
**Reviewer #1: The limitations of the study were not described explicitly.**

Response: A Limitation section has been added to the revision (lines 251-260).
**Reviewer #2: (No Response)**
--------------------

**Editorial and Data Presentation Modifications?**
**Reviewer #1: Minor suggestion to improve readability: (line 245) I suggest to perhaps switch the part of the sentence mentioning Wolbachia with the next sentence ("As more evidence..."). Because this is the first time Wolbachia was mentioned in the text, I think this part should be mentioned later as an example of strategies with accumulating evidence.**

Response: Thanks for the comment. We have adjusted this part accordingly (lines 244-248).
**Reviewer #2: Second sentence of introduction could be rewritten - "Unlike malaria, in recent years the number of deaths due to dengue continue to increase"**
Response: Amended as suggested (line 87). Thanks.  **Figure 1 should appear before in results before table 1.**

Response: Figure 1 appears in the Results section before Table 1 (lines 139-141).  **Conclusion line 253 - 262 could do with minor editing.**

Response: We have slightly edited the Conclusion section (lines 263-265; lines 269-271).
--------------------

**Summary and General Comments**
**Reviewer #1: The authors have performed a comprehensive search of articles and presented the results clearly. Minor suggestions were made to increase clarity in discussion part.**

Response: Thanks for the suggestions.
**Reviewer #2: (No Response)**

Best regards,

Zhiwei Xu (On behalf of all co-authors)
